# Impact of local therapy in metastatic renal cell carcinoma during medical treatment in a retrospective analysis

**DOI:** 10.1038/s41598-025-10926-x

**Published:** 2025-07-30

**Authors:** Sena Hoffer, Hendrik Eggers, Tabea Fröhlich, Paula Kappler, Maria-Luisa Tiemann, Viktor Grünwald, Christoph Henkenberens, Mohamed Omar, Robert M. Blach, Florian Heidel, Philipp Ivanyi

**Affiliations:** 1 Department of Hematology, Hemostasis, Oncology and Stem Cell Transplantation, Hannover Medical School, OE 6860, Carl-Neuberg Str. 1, Hannover, Germany; 2 Claudia Von Schilling Comprehensive Cancer Center Lower Saxony, Hannover, Germany; 3 Department of Trauma Surgery and Orthopedics, Hannover Medical School, Hannover, Germany; 4 Department of Radiotherapy, Hannover Medical School, Hannover, Germany; 5 Present Address: Interdisciplinary Genitourinary Oncology at the West-German Cancer Center, Clinic for Internal Medicine (Tumor Research) and Clinic for Urology, Essen University Hospital, Essen, Germany; 6 Present Address: Department of Hematology and Oncology, Klinikum Braunschweig, Braunschweig, Germany

**Keywords:** Renal cell carcinoma, Radiation therapy, Surgery, Palliative treatment, Multimodal treatment, Local therapy, Cancer, Urology, Outcomes research

## Abstract

Current guidelines on treatment of metastatic renal cell carcinoma (mRCC) suggest an emerging role of local therapy (LT). Still there is a lack of data which patients may benefit from additional LT once medical treatment (MT) is initiated. We retrospectively aim to characterize LT in patients with mRCC who underwent LT while receiving MT. 315/401 mRCC patients were eligible, thereof 163 (51.7%) received LT during MT (LT( +)), while 152 (48.3%) received only MT (LT(-)). Radiotherapy (49.1%) and surgery (41.7%) were the most frequently administered LT modalities. Overall survival (OS) was not superior in LT( +) vs. LT(-) (35.9, (95%-CI [confidence interval]: 29.8–42.0) vs. 20.3, (95%-CI: 10.3–30.3) months, log-rank p = 0.117). However, in a subgroup analysis the duration of MT prior to initiation of LT (≤ 6 months 24.1 (95%-CI: 18.6–29.6) vs. > 6 months: 43.0 (95%-CI: 32.2–36.2) months, log-rank p = 0.005) and the type of progression (oligoprogression: 44.0 (95%-CI: 31.5–56.5) vs. systemic progression: 29.6 (95%-CI: 23.4–35.8) months, log-rank p = 0.03) were associated with improved OS. We present the largest analysis of LT during MT. Our study has enhanced our understanding of LT utilization in mRCC after MT is already initiated. Ultimately, the inclusion of LT could improve OS in selected patients receiving MT.

## Introduction

Medical treatment of metastatic renal cell carcinoma (mRCC) has dramatically changed over the last two decades with the introduction of targeted therapies and checkpoint inhibitors (CPI)^1–4^. In contrast, higher evidence within the field of management of mRCC with local therapies (LT) barely gained additional evidence^5,6^. European guidelines, as well as the national German guidelines, primarily suggest LT with complete metastasectomy, or radiotherapy when favorable disease characteristics are present^5,6^. Although there is a lack of randomized controlled studies, there is a reliable amount of data showing a benefit of LT in carefully selected patients^5–7^. Of note, one systemic review on LT with 2350 patients consistently found improved overall survival (OS) and cancer specific survival after complete metastasectomy^7^. However, it appears that MT was not usually administered prior to LT or that in these cases little is known about the MT administered^5,6,8^.

In contrast to the aforementioned goal of improving OS with LT, there is a large number of patients with widespread mRCC under MT that receive LT for palliation and symptom relief. In mRCC with symptomatic metastasis or tumor progression, radiotherapy is deemed an effective treatment especially for bone and brain metastasis, while surgery is mostly considered the standard approach for oligo-metastasectomy, for pathologic fractures or treatment of spinal compression^5,6^.

In conclusion, guidelines and previous studies separately report on the efficacy of a local treatment, either before MT to achieve improved OS, or during MT for symptom relief. But there are only few studies on the efficacy of LT during MT that demonstrate a survival benefit. Looking at the overall picture of patients receiving LT while undergoing a sequence of MT, there is no reliable data on the effects of LT – either to characterize its use or to analyze its efficacy.

The aim of this study is to analyze OS in patients who underwent local therapy while receiving medical treatment (LT +) in comparison to those who received MT only (LT-).

### Patients and methods

#### Study design and data acquisition

This single center retrospective analysis included 315 out of 401 patients treated at our tertiary cancer center (Department of Hematology, Hemostasis, Oncology and Stem Cell Transplantation, Hannover Medical School, Hannover) for mRCC. Patients were identified within the observation period between 04/2000 −05/2016. Treatment data, patient characteristics and tumor features were evaluated retrospectively on the basis of medical records. The data was anonymized prior to storage and stored in the database of Microsoft Access before transferred into SPSS software platform (IBM) for statistical analysis. Inclusion criteria for this analysis were age > 18 years at diagnosis of metastasis, administration of at least one MT with palliative intent for mRCC. Patients with another neoplasia were excluded **(**Supplementary Table 1**)**. Tumor progression or response to treatment was defined by the treating physician based on clinical and/or radiographic evaluation. In this study, oligoprogression was defined as localized progression, i.e. progression of a single treated metastasis or several metastases in one organ system. Systemic progression refers to the progression of several metastases in at least two organ systems.

The ECOG performance status was assessed before and after LT and reported 3 months before or after LT, respectively. MT and LT, as well as the staging and supportive therapy were conducted according to local standard of care and applicable treatment guidelines as far as available. Local therapies consisted mainly of radiotherapy and surgical procedures **(**Table 2**)**. The latter were mainly used to perform metastasectomies, but also occasionally in the event of complications, for example to treat pathological fractures. Last follow up was performed on May 15, 2016.

Data handling and storage was performed by physicians and data managers in an anonymized manner and in accordance with the Helsinki Declaration in its latest version. Approval was granted by the local ethics committee (Hannover Medical School, Nr.: 3172–2016).

### Statistical analysis

Patients were grouped for comparison: LT(-) patients receiving only MT and no local therapy, LT(+) patients receiving MT and at least one additional LT. For group comparison we only compared the first local therapy. The Chi-square test, Mann–Whitney test, Fisher’s exact test and t-test were conducted as applicable. OS was calculated from initiation of first MT for mRCC until death or last follow up. Patients lost to follow-up were censored at the time of the last documented follow-up. Survival analysis was conducted by application of Kaplan–Meier analysis and log-rank test. The relationship between survival, tumor features and treatment characteristics was analyzed by univariate and multivariate analyses. Univariate analyses included all variables tested previously with p < 0.2 between LT(-) and LT(+). Multivariate analyses included parameters from univariate analysis with p < 0.2. A two-sided p-value below 0.05 was considered as statistically significant. SPSS was used for statistical analysis (IBM, V28, Armonk, New York).

## Results

### Patient demographics and tumor features

315 out of 401 patients treated for mRCC between April 2000 and May 2016 were eligible. Herein, 315 patients with MT were grouped in dependence of at least one administered LT during MT (LT(-): N = 152 (48.3%) and LT(+): N = 163 (51.7%)) **(**Supplementary Table 1**)**.

Overall, there was a predominance of male patients with diagnosis of mRCC and a median age of 61.4 years (median, range [r]: 26.4–89.6). Primary T-Stage were 3–4 (52.7%) and low grades (G1-2: 51.2%), with a predominance of clear cell histology (77.5%). 94.6% patients underwent previous nephrectomy. Patients within LT(+) were younger at diagnosis of metastasis (57.4, (r: 26.4–78.7) vs. 65.7, (r: 33.2–89.6) years, p < 0.05), showed favorable tumor grading (G1-2 56.4% vs. 45.3%, p < 0.05) and underwent nephrectomy more often than LT(-) patients (97.0% vs. 91.4%, p < 0.05) **(**Table 1**)**.

**Table 1:** Patient and tumor characteristics with characteristics and patterns of disease at diagnosis of mRCC stage.

	Total cohort N = 315 (100%)	LT(+) N = 163 (51.7%)	LT(-) N = 152 (48.3%)	p-value
**Sex**				0.96
Male, n (%)	218 (69)	113 (69.3)	105 (69.1)	
Female, n (%)	97 (31)	50 (30.7)	47 (30.9)	
**Age**				
**Primary diagnosis RCC**^i^ Age, median (range), years	58 (26–88)	54 (26–78)	61 (32–88)	< 0.05
**Diagnosis mRCC**^**j**^ Age, median (range), years	61.4 (26.4–89.6)	57.4 (26.4–78.7)	65.7 (33.2–89.6)	< 0.05
**Primary diagnosis RCC**				
T-Stage				0.25
1–2, n (%)	103 (32.7)	59 (36.2)	44 (28.9)	
3–4, n (%)	166 (52.7)	83 (50.9)	83 (54.7)	
NA, n (%)	46 (14.6)	21 (12.9)	25 (16.5)	
**Grading**				< 0.05
1–2, n (%)	161 (51.1)	92 (56.4)	69 (45.4)	
3–4, n (%)	101 (32.1)	45 (27.6)	56 (36.9)	
NA^c^, n (%)	53 (16.8)	26 (16)	27 (17.7)	
Clear cell RCC, n (%)	244 (77.5)	127 (78)	117 (77)	0.73
Nephrectomy^k^, n (%)	298 (94.6)	159 (97)	139 (91.4)	< 0.05
**Diagnosis mRCC – 1st MT**^**c**^ Time period, median (range), months	2.1 (0–167)	2.1 (0–97)	2 (0–167)	0.87
**Diagnosis mRCC – 1st LT**^**d**^ Time period, median (range)	17 (0–117)	18 (0–117)	NA^e^	
**ECOG**^**f**^				0.06
0–1, n (%)	227 (72.1)	124 (76.1)	103 (67.8)	
≥ 2, n (%)	14 (4.4)	4 (2.5)	10 (6.6)	
NA, n (%)	74 (23.5)	35 (21.4)	39 (25.6)	
**MSKCC**^**g**^				0.30
Favorable, n (%)	34 (10.8)	20 (2.5)	14 (9.2)	
Intermediate, n (%)	96 (30.5)	53 (32.5)	43 (28.3)	
Poor, n (%)	13 (4.1)	4 (12.3)	9 (5.9)	
NA, n (%)	172 (54.6)	86 (52.7)	86 (56.6)	
No. of metastasized organs, median, (range)^h^	2 (0–8)	1 (1–8)	2 (0–7)	< 0.05
Lung, n (%)	168 (53.3)	94 (57.7)	74 (48.7)	0.11
Bones, n (%)	90 (28.6)	45 (27.6)	45 (29.6)	0.69
Liver, n (%)	55 (17.5)	20 (12.3)	35 (23)	< 0.05
Lymph nodes, n (%)	106 (33.7)	55 (33.7)	51 (33.6)	0.97
Soft tissue, n (%)	27 (8.6)	15 (9.2)	12 (7.9)	0.68
Kidney, n (%)	32 (10.2)	12 (7.4)	20 (13.2)	0.09
Local recurrence, n (%)	28 (8.9)	9 (5.5)	19 (12.5)	< 0.05
Brain, n (%)	6 (1.9)	3 (1.8)	3 (2)	0.93
Number of MT, mean, range	2.7 (1–10)	3.2 (1–10)	2.2 (1–8)	< 0.05

**a)** Medical treatment + local therapy **b)** Medical treatment only **c)** Medical treatment **d)** Local therapy **e)** Not available **f)** Eastern Cooperative Oncology Group **g)** Memorial Sloan Kettering Cancer Center **h)** Multilocular organ metastases possible **i)** Renal cell carcinoma **j)** Metastatic renal cell carcinoma **k)** Cumulative number of primary partial/complete nephrectomy, as well as cytoreductive nephrectomies. Characteristics of the eligible cohort is displayed (total cohort), as well as those of the subgroups, either receiving additional local therapies (LT(+)) during medical treatment or not (LT(-)).

### Characteristics of metastatic disease

In the overall population, most patients showed a good performance status (ECOG 0–1: 72.1%) and an intermediate MSKCC risk score (30.5%), although many values were missing. Patients received 2.7 medical treatment lines (mean, r: 1–10) and the most common metastatic sites were lung (53.3%), bones (28.6%) and lymph nodes (33.7%).

In a subgroup analysis, LT(+) patients showed a better performance status (ECOG 0–1: 76.1% vs. 67.8%, p = 0.06) with similar MSKCC risk, but with less metastatic organ sites (median: 1, (r: 1–8) vs. 2, (r: 0–7), p < 0.05) and more lines of MT (mean: 3.2, (r: 1–10) vs. 2.2, (r: 1–8), p < 0.05). Interestingly, liver metastases were less common in LT(+) (median: 12.3% vs. 23%, p < 0.05), while lung metastases were equally distributed (median: 57.7% vs. 48.7%, p = 0.11) **(**Table 1**)**.

### Treatment characteristics

The most frequently administered LT was radiotherapy (49.1%), followed by surgery (41.7%). The most common metastatic sites treated with LT were bone (44.8%), brain (18.4%) and lung (12.9%). Progressive disease during MT leading to LT was considered as locally confined progression (oligoprogression) in 41.1% and as systemic progression in 42.9% of all patients with a total of 55.8% suffering from symptomatic burden.

The response to systemic treatment measured after LT was rather low and showed a partial response in 6.7% and a stable disease in 17.2%, but symptom relief was common, with 30.1% showing an improvement in symptoms **(**Table 2**)**. ECOG performance status was good before LT (median 0, (r: 0–4)) and declined after LT (median: 1, (r: 0–4)), but did not show a difference between surgery or radiotherapy and did also not decline in patients undergoing a sequence of local therapies **(**Supplementary Table 2**)**. MT mainly consisted of therapy targeting VEGF (vascular endothelial growth factor) in first (71.1%), second (53.5%) and third (56.9%) line therapy. CPI only accounted for 1.3%, 1.3% and 3.9% respectively, while cytokine therapy was still performed in first-, second-, and third-line therapy with 12.1%, 0.9% and 1.3% of patients, respectively **(**Supplementary Table 3**)**.

**Table 2:** Characteristics and patterns of first local therapy (LT).

	Total cohort N = 315 (100%)	LT(+)^a^ N = 163 (51.7%)	LT(-) N = 152 (48.3%)
**LT**^**b**^**, type**			
RTx^c^, n (%)	80 (49.1)	80 (49.1)	0 (0)
Surgery, n (%)	68 (41.7)	68 (41.7)	0 (0)
RFA^d^, n (%)	4 (2.5)	4 (2.5)	0 (0)
MWA^e^, n (%)	0 (0)	0 (0)	0 (0)
SIRT^f^, n (%)	2 (1.2)	2 (1.2)	0 (0)
Others, n (%)	9 (5.5)	9 (5.5)	0 (0)
**No. of MT**^**g**^			
Before LT, n, median (range)	1 (0–6)	1 (0–6)	NA^**l**^
After LT, n, median (range)	1 (0–7)	1 (0–7)	NA^**l**^
**Target organ of LT**			
Bones, n (%)	73 (44.8)	73 (44.8)	NA^**l**^
Kidney, n (%)	7 (4.3)	7 (4.3)	NA^**l**^
Lung, n (%)	21 (12.9)	21 (12.9)	NA^**l**^
Liver, n (%)	10 (6.1)	10 (6.1)	NA^**l**^
Lymph nodes, n (%)	18 (11)	18 (11)	NA^**l**^
Brain, n (%)	30 (18.4)	30 (18.4)	NA^**l**^
Skin, n (%)	3 (1.8)	3 (1.8)	NA^**i**^
Others^m^, n (%)	27 (16.6)	27 (16.6)	NA^**l**^
Total number of administered LT^h^, n	189	189	NA^**l**^
**Status before LT**			
Local PD, n (%)	67 (41.1)	67 (41.1)	NA^**l**^
Systemic PD, n (%)	70 (42.9)	70 (42.9)	NA^**l**^
NE^i^, n (%)	26 (16)	26 (16)	NA^**l**^
**Best systemic response after LT**			
Partial response, n (%)	11 (6.7)	11 (6.7)	NA^**l**^
Stable disease, n (%)	28 (17.2)	28 (17.2)	NA^**l**^
Progressive disease^j^, n (%)	80 (49.1)	80 (49.1)	NA^**l**^
Mixed response, n (%)	6 (3.7)	6 (3.7)	NA^**l**^
NE due to death, n (%)	20 (12.2)	20 (12.2)	NA^**l**^
NE, n (%)	18 (11.1)	18 (11.1)	NA^**l**^
**ECOG**^**k**^			
Before LT, Median (range)	0 (0–4)	0 (0–4)	NA^**l**^
After LT, Median (range)	1 (0–4)	1 (0–4)	NA^**l**^
**Clinical symptoms**			
**Before LT**			
Yes, n (%)	91 (55.8)	91 (55.8)	NA^**l**^
No, n (%)	29 (17.8)	29 (17.8)	NA^**l**^
NE, n (%)	43 (26.4)	43 (26.4)	NA^**l**^
**After LT**			
No change, n (%)	12 (7.4)	12 (7.4)	NA^**l**^
Improved, n (%)	49 (30.1)	49 (30.1)	NA^**l**^
Worsened, n (%)	16 (9.8)	16 (9.8)	NA^**l**^
NA^l^/NE n (%)	86 (52.7)	86 (52.7)	NA^**l**^

**a)** Medical treatment + local therapy **b)** Local therapy **c) **Radiotherapy **d)** Radiofrequency ablation** e)** Microwave ablation **f)** Selective internal radiation therapy **g) **Medical treatment **h) **20 patients had simultaneous multilocular local therapy **i) **Not evaluable **j) **Progressive disease (clinical and radiological judgement) **k) **Eastern Cooperative Oncology Group** l) **Not available **m)** Including all sites of metastases other than those mentioned above and sites, that have not been described in detail.

### Outcome

**Fig. 1:**
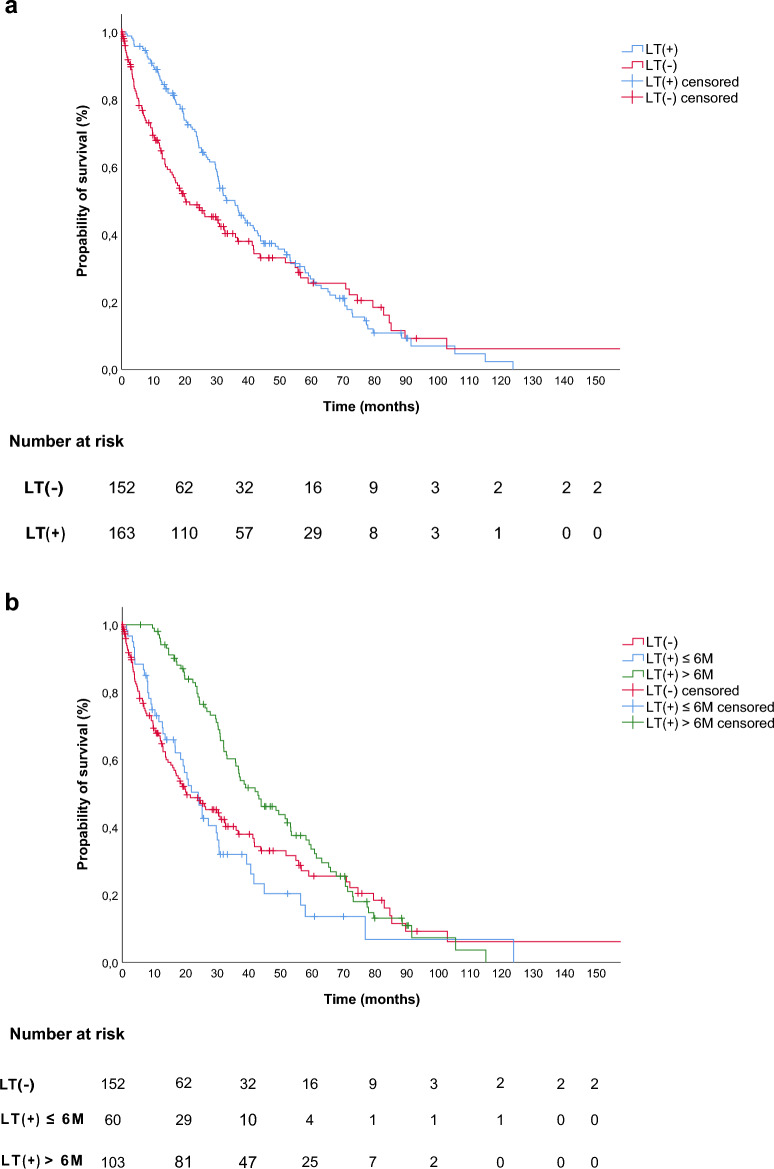
Overall survival in all mRCC patients with and without local treatment (LT(+); LT(-)) initiated during medical treatment **a**) OS of LT(+) vs. LT(-) 35.9 (95%-CI: 29.8–42.0) vs. 20.3, (95%-CI: 10.3–30.3) months, log-rank p = 0.117). **b)** OS in of LT(+) vs. LT(-) in dependence of LT initiation, either within 6 months (LT(+) ≤ 6 M), or beyond 6 months (LT(+) > 6 M) after initiation of medical treatment (LT(-): 20.3 (95%-CI: 10.3–30.3) vs. LT(+) ≤ 6 M: 24.1 (95%-CI: 18.6–29.6) vs. LT(+) > 6 M: 43.0 (95%-CI 32.2–36.2) months, log-rank p = 0.005).

With a follow-up period of 24 months, the median OS in the total study population was 30.7 months (95%-CI: 25.2–36.2 months). Although the median OS in LT(+) was numerically higher compared to LT(-), the difference was not significant (35.9 (95%-CI: 29.8–42.0) vs. 20.3 (95%-CI: 10.3–30.3) months, p = 0.117, log-rank **(**Fig. 1a**)**). No difference was identified in OS between LT(+) and LT(-) in the subgroup of patients receiving LT within 3, 6 or 9 months after starting MT (data not shown). However, a comparison of LT(-) with patients who received LT within 6 months and later than 6 months, revealed a significant improvement in the median OS for patients with late LT (LT(-): 20.3 (95%-CI: 10.3–30.3) vs. LT(+) ≤ 6 M: 24.1 (95%-CI: 18.6–29.6) vs. LT(+) > 6 M: 43.0 (95%-CI 32.2–36.2) months, p = 0.005, log-rank **(**Fig. 1b**)**).

**Fig. 2:**
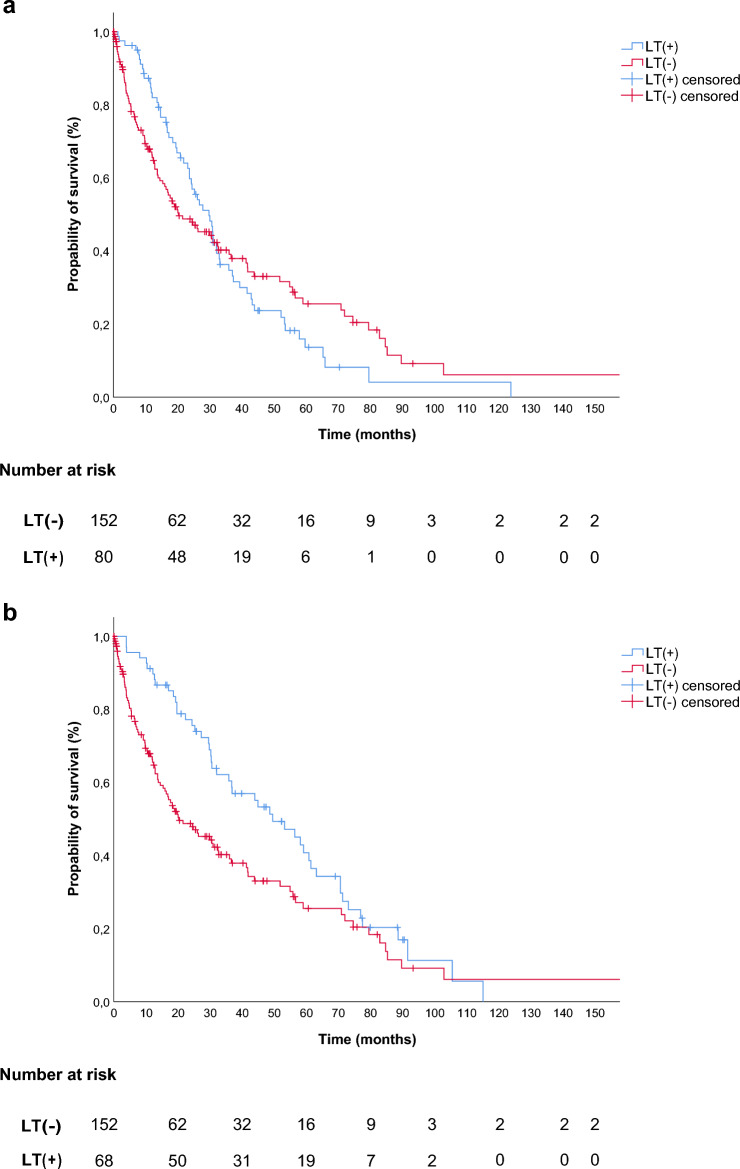

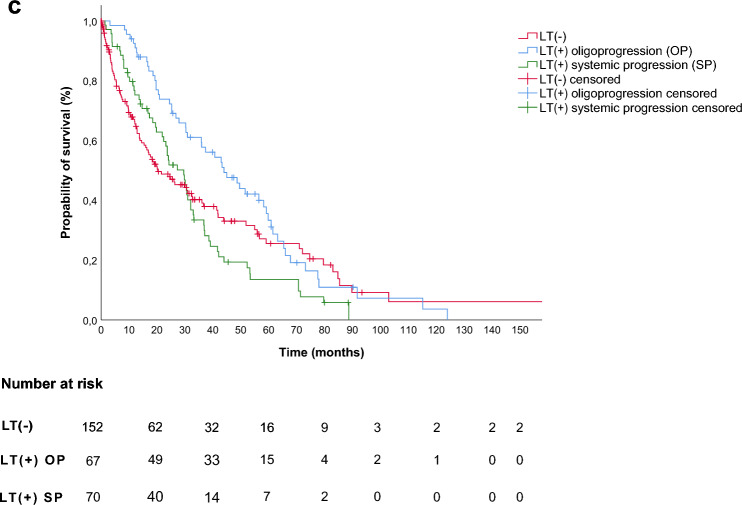
Overall survival in subgroups in dependence of the administered LT modality **a**) LT(+) of patients receiving radiotherapy as first LT(+) vs. LT(-) (LT(+): 29.8 (95%-CI: 24.2–35.4) vs. LT(-): 20.3 (95%-CI: 10.3–30.3) months, log-rank, p = 0.89). **b)** LT(+) of patients receiving surgery as first local therapy vs. LT(-) (LT(+): 49.6 (95%-CI: 34.7–64.5) vs. (LT(-): 20.3 (95%-CI: 10.3–30.3) months, log-rank p = 0.01). **c)** LT(-) vs. LT(+) with patients defined by local progression of mRCC vs. LT(+) defined by systemic progression of mRCC (LT(-): 20.3 (95%-CI: 10.3–30.3) vs. LT(+) local PD: 44 (95%-CI: 31.5–56.5) vs. (LT(+) systemic PD: 29.6 (95%-CI: 23.4–35.8) months, log-rank p = 0.03).

In the subgroup analysis of patients with radiotherapy vs. LT(-) there was no difference in the median OS (29.8 (95%-CI: 24.2–35.4) vs. 20.3 (95%-CI: 10.3–30.3) months, p = 0.89, log-rank **(**Fig. 2a**)**). There was an improved median OS for patients with surgery compared to LT(-) (49.6 (95%-CI: 34.7–64.5) vs. 20.3 (95%-CI: 10.3–30.3) months, p = 0.01, log-rank **(**Fig. 2b**)**).

Further analysis of LT(-) compared to LT(+) diagnosed with either local or systemic disease progression also revealed an association of improved OS with LT for local disease progression i.e. oligoprogression (20.3 (95%-CI: 10.3–30.3) vs. 44.0 (95%-CI: 31.5–56.5) vs. 29.6 (95%-CI: 23.4–35.8) months, p = 0.03, log-rank **(**Fig. 2c**)**).

### Univariate and multivariate analysis

In univariate analysis tumor grading, nephrectomy, ECOG, the number of metastasized organs, the application of radiotherapy, the application of LT before or after 6 months, the type of progression before LT, number of MT, the presence of lung metastases and the presence of a clear cell histology was associated with a significant impact on overall survival. In multivariate analysis the application of LT after 6 months, a local progression before LT (i.e. oligoprogression), an administered MT above mean and the presence of a clear cell histology was associated with improved overall survival **(**Supplementary Tables 4 and 5**)**.

## Discussion

This retrospective single center analysis was designed to elucidate the characteristics and efficacy of LT within the context of sequential MT of mRCC comparing the outcome in LT(+) and LT(-) patients.

Overall, 315 patients were included with baseline characteristics showing mostly a typical mRCC population^5^. While patients were notably young (median 58 years), patients were even younger in the LT(+) group (median 54 vs. 61 years). Therefore, young age, as well as low tumor grading in LT(+), possibly reflect a plausible biological selection process in the utilization of LT. Nevertheless, there was no difference in ECOG performance status, or MSKCC risk score between LT(+) and LT(-), although risk assessment was insufficient in our cohort. LT(+) patients received more lines of MT, while MT was mainly VEGF-based reflecting the standard of care during our observation period. Only 1.3% of patients received CPI as first line therapy, meaning all findings should be interpreted within the context of a shift towards CPI-based therapy as the current standard of care. Our results cannot be generalized.

Radiotherapy (49.1%) and surgery (41.7%) were the most common administered local therapies during MT, and a broad variety of metastatic locations was addressed. Looking at tumor response it must be noted that 41.1% showed a localized progression, clinically most likely to be regarded as oligoprogression during MT prior to LT. Best systemic response was expectably low (6.7%), but symptomatic relief frequent (30.1%). In summary, response to LT shows some benefit in selected patients within a mixed cohort defined by type and localization of LT.

When assessing treatment results, our study revealed a median OS of 30.7 months comparable to the pivotal phase 3 trial of pazopanib vs. sunitinib with 28.3, respectively 29.1 months, reflecting the era dominated by VEGFR-inhibition^9^. This finding reflects the observation period. Most importantly, there was no clear OS benefit for all LT(+) patients, but a notable trend was observed and further analysis identified subgroups with a substantial benefit of LT during sequential MT. Firstly, an association with improved OS was found in patients who underwent LT later than six months after starting MT. Secondly, patients with localized disease progression showed improved OS with the use of LT. Consequently, we would conclude that patients who achieved a durable tumor control through MT and showed limited disease progression – especially oligoprogression – were most likely to benefit from LT in terms of OS. Both findings are additionally supported by multivariate analysis, while LT(+) patients with surgery and radiotherapy failed to show a significant benefit.

In general, a benefit of metastasectomy postponing initiation of MT and improving OS has been widely accepted^7^. However, incorporation of LT or metastasectomy into sequential MT has rarely been studied before^10,11^. The only study similar to ours, but much smaller, compared 75 patients with mRCC and MT. 26 of these patients had a complete metastasectomy and 23 had an incomplete metastasectomy after at least one targeted therapy. Herein, a notable OS benefit was associated with complete metastasectomy vs. MT only (5.1 vs. 2.4 years), but many aspects outlined in our study were not covered^10^.

There is a limited number of studies on radiotherapy that indicate a benefit of local radiotherapy in oligo-progressive mRCC^12–14^. Although none of these studies includes a comparator arm, all of them show an exceptional rate of local tumor control (above 90%) and a progression free survival of 9 to 10 months without a change in MT. This data may support our notion that LT may not only delay the necessity for a change in MT, but could also be suitable for improving the outcome for the patients. This hypothesis is further supported by a retrospective analysis of 55 patients treated with radiotherapy for oligo-progressive disease showing an OS benefit when continuing MT after radical LT^15^.

Summarizing previous studies on surgery and radiotherapy in the context of MT, some patients seem to benefit from a combined approach in regard to improved OS. This ultimately supports the current guideline recommendations, which suggest an emerging role of LT in oligoprogression and highlight the importance of our findings^5^. In addition, our results emphasize the importance of patient selection by timing and type of progression, rather than demonstrating a general benefit of LT or any type of LT.

While the nature of our analysis does not allow a distinct conclusion on the underlying mechanism, it can be assumed that patients with a better response to MT also benefit from LT. In an analysis of Santini et al. they were able to show an advantage in terms of disease-free survival when complete remission was achieved with systemic therapy in combination with local therapy compared to systemic therapy alone^16^. Nevertheless, we assume that a generally more favorable tumor biology is associated with oligoprogression and later use of LT after the start of MT. Whilst this may not be surprising, as far as we know there is no comparable data on the timing of LT use compared to MT.

As far as we know, this is the most comprehensive study analyzing the effects of LT during MT. We provide low evidence for a benefit of LT in patients during MT for mRCC. Patients characterized by the duration of MT (> 6 months) prior to LT and most importantly by type of progression (oligoprogression), might benefit the most, very likely due to an indolent disease course and a distinct tumor biology. Therefore, we propose to consider LT for the treatment of selected patients who fulfil these criteria during MT.

Limitations of our study are its retrospective nature and a significant selection bias including a higher proportion of beneficial disease characteristics in the LT(+) group. The latter is made more difficult to assess by the lack of data in the MSKCC documentation. Our results should therefore be regarded as hypothesis-generating. Moreover, due to the selected observation period only few patients received CPI or a combination therapy. Hence, our study does not reflect the current standard of care. However, we do believe that the analysis of a large cohort, undergoing sequential treatment with VEGF-inhibition, mTOR-inhibition, cytokines and CPI, adds substantial knowledge to our understanding of LT during MT in mRCC, especially as VEGFR-inhibition still is a pillar of therapy. Further analyses are warranted as additive local therapy could provide significant survival benefits when administered to appropriate patients.

## Supplementary Information

Supplementary Information.

## Data Availability

Data is provided within the manuscript and the supplementary information. The datasets generated during the current study are available from the corresponding authors on reasonable request.

## References

[1] Motzer, R. J. et al. Sunitinib versus interferon alfa in metastatic renal-cell carcinoma. *N Engl J Med* **356**, 115–124. 10.1056/NEJMoa065044 (2007). 17215529 10.1056/NEJMoa065044

[2] Motzer, R. J. et al. Nivolumab versus Everolimus in Advanced Renal Cell Carcinoma. *N Engl J Med* **373**, 1803–1813. 10.1056/NEJMoa1510665 (2015). 26406148 10.1056/NEJMoa1510665 PMC5719487

[3] Motzer, R. J. et al. Nivolumab plus Ipilimumab versus Sunitinib in Advanced Renal-Cell Carcinoma. *N. Engl. J. Med.* **378**, 1277–1290. 10.1056/NEJMoa1712126 (2018). 29562145 10.1056/NEJMoa1712126 PMC5972549

[4] Rini, B. I. et al. Pembrolizumab plus Axitinib versus Sunitinib for Advanced Renal-Cell Carcinoma. *N. Engl. J. Med.* **380**, 1116–1127. 10.1056/NEJMoa1816714 (2019). 30779529 10.1056/NEJMoa1816714

[5] Escudier, B. et al. Renal cell carcinoma: ESMO Clinical Practice Guidelines for diagnosis, treatment and follow-up. *Ann. Oncol.* **30**, 706–720. 10.1093/annonc/mdz056 (2019). 30788497 10.1093/annonc/mdz056

[6] Deutsche Krebsgesellschaft, Deutsche Krebshilfe, AWMF Leitlinienprogramm Onkologie (Deutsche Krebsgesellschaft, Deutsche Krebshilfe, AWMF): S3-Leitlinie Diagnostik, Therapie und Nachsorge des Nierenzellkarzinoms, Langversion 4.0, 2023, AWMF-Registernummer: 043–017OL https://www.leitlinienprogramm-onkologie.de/leitlinien/nierenzellkarzinom/; accessed 11.07.2023 (2023).

[7] Dabestani, S. et al. Local treatments for metastases of renal cell carcinoma: A systematic review. *Lancet Oncol.* **15**, e549-561. 10.1016/S1470-2045(14)70235-9 (2014). 25439697 10.1016/S1470-2045(14)70235-9

[8] Ivanyi P, Kuczyk M [Synchronous oligometastatic renal cell carcinoma-what is the role of surgery?]. Urol Ausg A 60:1546–1554. Robert Koch Institute (ed.) and the Association of Population-based Cancer Registries in Germany (2021) Cancer in Germany 2017/2018. 158 10.1007/s00120-021-01700-89 (2021). 10.1007/s00120-021-01700-8 34738151

[9] Motzer, R. J. et al. Pazopanib versus sunitinib in metastatic renal-cell carcinoma. *N Engl J Med* **369**, 722–731. 10.1056/NEJMoa1303989 (2013). 23964934 10.1056/NEJMoa1303989

[10] Li, J.-R. et al. The Impact of Local Intervention Combined with Targeted Therapy on Metastatic Renal Cell Carcinoma. *Anticancer Res.* **38**, 5339–5345. 10.21873/anticanres.12861 (2018). 30194186 10.21873/anticanres.12861

[11] Mikhail, M., Chua, K. J., Khizir, L., Tabakin, A. & Singer, E. A. Role of metastasectomy in the management of renal cell carcinoma. *Front. Surg.* **29**(9), 943604. 10.3389/fsurg.2022.943604 (2022). 10.3389/fsurg.2022.943604 PMC9372304 35965871

[12] Cheung, P. et al. Stereotactic Radiotherapy for Oligoprogression in Metastatic Renal Cell Cancer Patients Receiving Tyrosine Kinase Inhibitor Therapy: A Phase 2 Prospective Multicenter Study. *Eur. Urol.* **80**, 693–700. 10.1016/j.eururo.2021.07.026 (2021). 34399998 10.1016/j.eururo.2021.07.026

[13] De, B. et al. Definitive Radiotherapy for Extracranial Oligoprogressive Metastatic Renal Cell Carcinoma as a Strategy to Defer Systemic Therapy Escalation. *BJU Int.* **129**, 610–620. 10.1111/bju.15541 (2022). 34228889 10.1111/bju.15541 PMC10097479

[14] Hannan, R. et al. Phase II Trial of Stereotactic Ablative Radiation for Oligoprogressive Metastatic Kidney Cancer. *Eur. Urol. Oncol.* **5**, 216–224. 10.1016/j.euo.2021.12.00 (2022). 34986993 10.1016/j.euo.2021.12.001 PMC9090939

[15] Santini, D. et al. Outcome of oligoprogressing metastatic renal cell carcinoma patients treated with locoregional therapy: A multicenter retrospective analysis. *Oncotarget* **8**, 100708–100716. 10.18632/oncotarget.2002 (2017). 29246014 10.18632/oncotarget.20022 PMC5725056

[16] Santini, D. et al. Risk of recurrence and conditional survival in complete responders treated with TKIs plus or less locoregional therapies for metastatic renal cell carcinoma. *Oncotarget* **7**(22), 33381. 10.8632/oncotarget.8302 (2016). 27027342 10.18632/oncotarget.8302 PMC5078103

