# Supplementary material for: Impact of local therapy in metastatic renal cell carcinoma during medical treatment in a retrospective analysis
Source: Sci Rep. 2025 Jul 30;15:27843. doi: 10.1038/s41598-025-10926-x (PMC12310947; doi:10.1038/s41598-025-10926-x)
Supplement: Supplementary file 1 — Supplementary Information. [file 41598_2025_10926_MOESM1_ESM.pdf]

Supplementary Information

Article title: Impact of local therapy in metastatic renal cell carcinoma during medical treatment in a retrospective analysis

Journal: Scientific Reports

Authors:

*Sena Hoffer [1,2 §] ,Hendrik Eggers [1,2,8 §], Tabea Fröhlich [1,2], Paula Kappler [1,2], Maria-Luisa Tiemann [1,2],

Viktor Grünwald [1,3,4,5], Christoph Henkenberens [7], Mohamed Omar [2,6], Robert M. Blach [2,7],

Florian H. Heidel [1,2], Philipp Ivanyi [1,2,3,5]

Affiliation:

[1] Department of Hematology, Hemostasis, Oncology and Stem Cell Transplantation, Hannover Medical School, Hannover, Germany

[2] Claudia von Schilling Comprehensive Cancer Center Lower Saxony, Hannover, Germany

[3] Interdisciplinary Working Group Renal Cell Cancer of German Cancer Society (IAGN-DKG)

[4] Current affiliation: Interdisciplinary Genitourinary Oncology at the West-German Cancer Center, Clinic for Internal Medicine (Tumor research) and Clinic for Urology, Essen University Hospital, Essen, Germany

[5] GUARDIAN: GenitoUrinary cAncer Research group on Development and Innovations in patients with Advanced maligNancieS

[6] Department of Trauma Surgery and Orthopedics, Hannover Medical School, Hannover, Germany

[7] Department of Radiotherapy, Hannover Medical School, Hannover, Germany

[8] Current affiliation: Department of Hematology and Oncology, Klinikum Braunschweig, Braunschweig, Germany

§ Equal contribution

***Corresponding Authors:**

PD Dr. med. Philipp Ivanyi, email: Ivanyi.philipp@mh-hannover.de
Sena Hoffer, email: Hoffer.sena@mh-hannover.de

Supplementary Table 1

CONSORT diagram for the disposition of patients

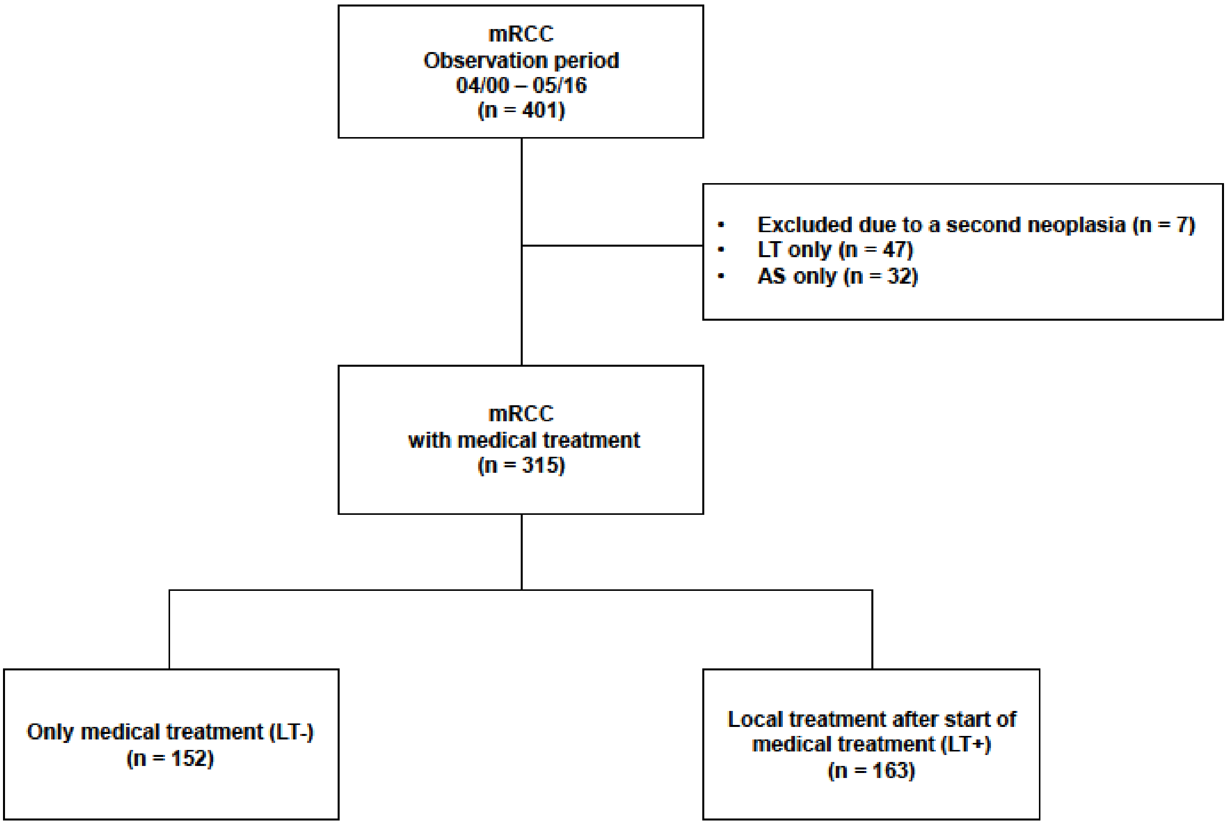

mRCC = Metastatic renal cell carcinoma, AS = Active surveillance

Supplementary Table 2

Characteristics of the performance status during sequential LT

	**LT1[a] N = 163 (100%)**	**LT2 N = 95 (100%)**	**LT3 N = 57 (100%)**
**No. of administered radiotherapies**	80 (49.1)	56 (34.4)	31 (19)
**ECOG[b] before RTx[c]**, median (range)	0 (0-4)	0 (0-3)	1 (0-1)
**ECOG after RTx**, median (range)	1 (0-4)	1 (0-4)	1 (0-3)
**No. of administered surgeries**	68 (41.7)	34 (20.9)	22 (13.5)
**ECOG before surgery**, median (range)	0 (0-4)	0 (0-4)	0 (0-4)
**ECOG after surgery**, median (range)	1 (0-4)	1 (0-4)	1 (0-4)

a) Local therapy **b)** Eastern Cooperative Oncology Group **c)** Radiotherapy

Supplementary Table 3

Characteristics of sequential medical treatment lines

Type of Medication	1stL[a], n=315	2ndL[b], n=228	3rdL[c], n=153
**VEGF**, n (%)	224 (71.1)	122 (53.5)	87 (56.9)
**mTOR**, n (%)	13 (4.1)	84 (36.8)	48 (31.4)
**Cytokine**, n (%)	38 (12.1)	2 (0.9)	2 (1.3)
**Cytokine + Chemo**, n (%)	24 (7.6)	4 (1.8)	1 (0.6)
**Checkpoint**, n (%)	4 (1.3)	3 (1.3)	6 (3.9)
**Chemo**, n (%)	3 (0.9)	1 (0.4)	7 (4.6)
**Others**, n (%)	9 (2.9)	12 (5.3)	2 (1.3)

a) First-line treatment **b)** Second-line treatment **c)** Third-line treatment

Supplementary Table 4

Univariate and multivariate analyses

**Prognostic factor**	**Survival**<br><br>**Univariate (HR 95%-CI)**	**p-value**	**Multivariate (HR 95%-CI)**	**p-value**
**Alter**				
≤ 60 years	1			
> 60 years	1.067 (0.82-1.39)	0.63		
**Grading**				
G1 + G2	1		1	
G3 + G4	1.59 (1.18-2.14)	0.002	1.10 (0.65-1.88)	0.72
**Nephrectomy**				
No	1		1	
Yes	0.42 (0.24-0.74)	0.003	0.19 (0.02-1.67)	0.13
**ECOG**				
0-1	1		1	
2-3	5,71 (3.03-10.78)	<0.001	0.86 (0.31-4.03)	0.86
**No. of metastasized organs**				
0-1	1		1	
≥2	1.44 (1.10-1.88)	0.007	0.95 (0.56-1.60)	0.84
**Local therapy**				
No	1			
Yes	1.01 (0.73-1.83)	0,96		
**Local therapy (surgery)**				
No	1		1	
Yes	0.82 (0.62-1.08)	0.16	0.74 (0.33-1.64)	0.45
**Local therapy (radiotherapy)**				
No	1		1	
Yes	1.57 (1.16-2.11)	0.003	1.74 (0.79-3.84)	0.17
**Local therapy**				
≤ 6 months after MT[a]	1		1	
> 6 months after MT[a]	0.51 (0.35-0.74)	<0.001	0.47 (0.28-0.78)	0.003

Univariate and multivariate analyses

**Prognostic factor**	**Survival**<br><br>**Univariate (HR 95%-CI)**	**p-value**	**Multivariate (HR 95%-CI)**	**p-value**
**Type of progression before LT[b]**				
Local	1		1	
Systemic	1.79 (1.22 -2.63)	0.003	1.891 (1.18-3.01)	0.008
**Number of MT[a]**				
>mean	1		1	
<mean	0.50 (0.39-0.66)	<0.001	0.40 (0.23-0.68)	<0.001
**Lung metastasis**				
No	1		1	
Yes	1.34 (1.02-1.75)	0.03	1.43 (0.84-2.43)	0.19
**Liver metastasis**				
No	1		1	
Yes	1.18 (0.82-1.70)	0.36	1.73 (0.65-4.61)	
**Local recurrence**				
No	1			
Yes	1.38 (0.86-2.21)	0.19		0.27
**Kidney metastasis**				
No	1		1	
Yes	1.21 (0.80-1.84)	0.37	2.08 (1.07-4.03)	
**Histology**				
Clear Cell	1			
Non-Clear Cell	1.85 (1.27-2.70)	0.001		0.03
**MSKCC**				
fav. vs. int./poor				
favorite	1			
intermediate/poor	1.97 (1.25-3.11)	0.004		
fav. vs. not available				
favorite	1			
not available	1.20 (0.77-1.88)	0.42		
int./poor. vs. not available				
int./poor	1			
not available	0.57 (0.43-0.76)	<0.001		

a) Medical treatment **b)** Local therapy

All items included with significant differences in previous testing and items of interest; MSKCC not included due to a vast number of missing values; multivariate performed for significant findings in univariate analysis with p<0.20

Supplementary Table 5

Univariate and multivariate analyses including MSKCC

**Prognostic factor**	**Survival**<br><br>**Univariate (HR 95%-CI)**	**p-value**
**Alter**		
≤ 60 years	1	
> 60 years	1.067 (0.82-1.39)	0.63
**Grading**		
G1 + G2	1	
G3 + G4	1.59 (1.18-2.14)	0.002
**Nephrectomy**		
No	1	
Yes	0.42 (0.24-0.74)	0.003
**ECOG**		
0-1	1	
2-3	5,71 (3.03-10.78)	<0.001
**No. of metastasized organs**		
0-1	1	
≥2	1.44 (1.10-1.88)	0.007
**Local therapy**		
No	1	
Yes	1.01 (0.73-1.83)	0,96
**Local therapy (surgery)**		
No	1	
Yes	0.82 (0.62-1.08)	0.16
**Local therapy (radiotherapy)**		
No	1	
Yes	1.57 (1.16-2.11)	0.003
**Local therapy**		
≤ 6 months after MT[a]	1	
> 6 months after MT[a]	0.51 (0.35-0.74)	<0.001

Univariate and multivariate analyses including MSKCC

Prognostic factor	Survival<br>Univariate<br>(HR 95%-CI)	p-value
**Type of progression before LT[b]**		
Local	1	
Systemic	1.79 (1.22 -2.63)	0.003
**Number of MT[a]**		
>mean	1	
<mean	0.50 (0.39-0.66)	<0.001
**Lung metastasis**		
No	1	
Yes	1.34 (1.02-1.75)	0.03
**Liver metastasis**		
No	1	
Yes	1.18 (0.82-1.70)	0.36
**Local recurrence**		
No	1	
Yes	1.38 (0.86-2.21)	0.19
**Kidney metastasis**		
No	1	
Yes	1.21 (0.80-1.84)	0.37
**Histology**		
Clear Cell	1	
Non-Clear Cell	1.85 (1.27-2.70)	0.001
**MSKCC**		
fav. vs. int./poor		
favorite	1	
intermediate/poor	1.97 (1.25-3.11)	0.004
fav. vs. not available		
favorite	1	
not available	1.20 (0.77-1.88)	0.42
int./poor. vs. not available		
int./poor	1	
not available	0.57 (0.43-0.76)	<0.001

a) Medical treatment **b)** Local therapy

All items included with significant differences in previous testing and items of interest; multivariate performed for significant findings in univariate analysis with p<0.05 was incalculable.
